# Optimizing Pain Management in Emergency Departments: A Comprehensive Review of Current Analgesic Practices

**DOI:** 10.7759/cureus.69789

**Published:** 2024-09-20

**Authors:** Anmol K Nagpal, Charuta Gadkari, Akhilesh Singh, Aditya Pundkar

**Affiliations:** 1 Emergency Medicine, Jawaharlal Nehru Medical College, Datta Meghe Institute of Higher Education and Research, Wardha, IND; 2 Orthopedics, Jawaharlal Nehru Medical College, Datta Meghe Institute of Higher Education and Research, Wardha, IND

**Keywords:** emergency departments, multimodal approaches, non-pharmacologic interventions, pain assessment, pain management, pharmacologic interventions

## Abstract

Effective pain management in Emergency Departments (EDs) is vital for improving patient comfort and clinical outcomes. This review provides a comprehensive analysis of current pain management practices in ED settings, focusing on the challenges and opportunities for optimization. The review examines pharmacologic and non-pharmacologic pain management strategies, evaluating their effectiveness and identifying inconsistencies and gaps in current practices. Key challenges in the ED environment include time constraints, variability in clinical protocols, and the need to address diverse patient needs, including those of paediatric, geriatric, and chronic pain patients. The review highlights the importance of standardized pain assessment tools and protocols to improve consistency in pain management. Innovations, such as technological advances and multimodal approaches, are explored for their potential to enhance pain management practices. Recommendations address identified challenges, including improved training for ED staff, the development of evidence-based protocols, and the integration of multimodal pain management strategies. By addressing these areas, the review aims to contribute to the development of more effective and uniform pain management practices in emergency care, ultimately leading to better patient outcomes and experiences. This review emphasizes the need for ongoing research and adaptation of best practices to meet the evolving needs of patients in emergency settings.

## Introduction and background

Pain management is a fundamental aspect of care in Emergency Departments (EDs), where patients frequently present with acute and often severe pain stemming from a variety of causes [1]. The primary objective in these high-pressure environments is to provide rapid and effective relief to enhance patient comfort and facilitate prompt recovery. Effective pain management not only alleviates suffering but also plays a crucial role in improving overall clinical outcomes [2]. Properly managed pain can mitigate the psychological stress and anxiety associated with acute injuries and illnesses, which can, in turn, support quicker physical recovery and enhance the patient’s overall experience in the ED [3].

Despite its importance, pain management in the ED setting is fraught with challenges. The fast-paced nature of emergency care, characterized by high patient volumes and diverse case presentations, imposes significant time constraints on medical staff [4]. These constraints can limit the depth of pain assessment and the ability to implement comprehensive pain management strategies [5]. Furthermore, the variability in clinical practices and protocols among different EDs can lead to inconsistencies in assessing and managing pain. These challenges necessitate a thorough examination of current practices to identify potential areas for improvement and ensure that patients receive the best possible care [5].

This review aims to provide a detailed and comprehensive analysis of pain management practices in EDs. It seeks to achieve several key goals. First, it aims to assess current practices by evaluating the effectiveness of existing pain management strategies, including pharmacologic and non-pharmacologic interventions applied in the ED setting. By examining these practices, the review will shed light on the strengths and weaknesses of current approaches.

## Review

Overview of pain management strategies

Pain management strategies can be broadly divided into pharmacologic and non-pharmacologic interventions, emphasizing multimodal approaches that integrate various methods for enhanced outcomes [6]. Pharmacologic interventions primarily consist of analgesics, which are categorized into three main types: opioids, non-opioids, and adjuvants. Opioids are effective for managing moderate to severe pain, particularly in acute settings [7]. However, their use is associated with significant risks, including addiction and various adverse effects, necessitating careful consideration and monitoring. Non-opioid analgesics, such as nonsteroidal anti-inflammatory drugs (NSAIDs) and acetaminophen, are typically the first-line treatments for mild to moderate pain due to their more favourable safety profile compared to opioids [8,9]. Research indicates that multimodal analgesia - combining different classes of analgesics - can offer superior pain relief while minimizing side effects associated with higher doses of individual medications. Combining NSAIDs with opioids can enhance overall pain control and allow for reduced opioid doses, thus decreasing the risk of opioid-related adverse effects [10]. Non-pharmacologic interventions are also integral to pain management. Physical therapy aids in managing pain through movement and rehabilitation, particularly for chronic pain conditions. Cognitive-behavioural therapy (CBT) is another effective method, helping patients manage pain by altering their perceptions and responses to pain stimuli [11]. Multimodal pain management emphasizes the use of multiple analgesic agents that target different pain pathways. This strategy aims to improve pain relief and reduce reliance on opioids. By integrating pharmacologic and non-pharmacologic interventions, healthcare providers can tailor pain management strategies to individual patient needs, enhancing efficacy and safety [10]. Evidence suggests that multimodal approaches lead to better pain control, lower opioid consumption, and reduced incidence of side effects. For instance, combining regional anaesthesia with systemic analgesics has been shown to decrease postoperative pain and opioid use in surgical patients [10]. Furthermore, a multimodal approach allows for personalization based on the patient’s pain profile, preferences, and clinical situation, ultimately improving patient satisfaction and outcomes [10]. An overview of pain management strategies in emergency care is provided in Table 1.

**Table 1:** Overview of pain management strategies in emergency care NSAIDs: Non-steroidal anti-inflammatory drugs

Pain management strategy	Description	Common medications/techniques	Indications	Advantages	Limitations
Pharmacological management [9]	Use of medications to alleviate pain through various mechanisms such as NSAIDs, opioids, and local anaesthetics.	NSAIDs (e.g., ibuprofen, naproxen); opioids (e.g., morphine, fentanyl); local Anaesthetics (e.g., lidocaine)	Acute and chronic pain	Quick relief, variety of drugs for different pain levels	Risk of side effects, dependency (opioids), overdose risks
Non-pharmacological management [12]	Non-drug interventions to control pain, focusing on behavioural or physical therapy methods	Cognitive-behavioural therapy (CBT), physical therapy, acupuncture, TENS (transcutaneous electrical nerve stimulation)	Chronic pain, adjunct to pharmacological treatment	No drug-related side effects, long-term pain management	It may require multiple sessions, not immediately effective
Multimodal analgesia [13]	Combination of different analgesics or interventions to target pain via multiple pathways	NSAIDs + opioids, local anaesthetics + NSAIDs, physical therapy + pharmacological interventions	Acute postoperative pain, severe trauma	Synergistic effect, reduced doses of opioids, improved outcomes	Requires careful coordination, higher complexity in management
Patient-controlled analgesia (PCA) [14]	Allows patients to self-administer preset doses of pain medication, typically opioids, within safety limits	IV morphine, fentanyl, hydromorphone	Postoperative pain, severe acute pain	Provides autonomy to patients, more immediate pain relief	Risk of overuse, potential opioid side effects
Regional anaesthesia [15]	Injection of local anaesthetics near nerves to block pain in specific areas, often used during surgery or for chronic pain	Epidural anaesthesia, peripheral nerve blocks, spinal anaesthesia	Surgical pain, labour pain, chronic localized pain	Effective localized pain control avoids systemic side effects of opioids	Requires specialized skills, risk of nerve damage
Sedation and Anaesthesia [16]	Use of sedation or general anaesthesia to manage pain and anxiety, especially in severe acute cases or during procedures	Propofol, ketamine, midazolam	Severe acute pain, procedural pain	Excellent control over severe pain reduces anxiety	Requires close monitoring, potential respiratory and cardiovascular complications
Topical analgesia [17]	Use creams, patches, or gels with pain-relieving properties applied directly to the skin	Lidocaine patches, capsaicin cream, diclofenac gel	Localized pain, neuropathic pain, minor injuries	Minimally invasive, localized effect without systemic side effects	Limited effectiveness for severe or deep pain

Current practices in pain management

Pain management strategies are typically categorized into pharmacologic and non-pharmacologic approaches, with an increasing emphasis on multimodal methods that integrate various techniques to improve outcomes [6]. Pharmacologic interventions primarily involve the use of analgesics, which are classified into three major categories: opioids, non-opioids, and adjuvants. Opioids are effective for managing moderate to severe pain, particularly in acute settings, but their use carries significant risks, including addiction and adverse effects, requiring careful monitoring and consideration [6]. Non-opioid analgesics, such as NSAIDs and acetaminophen, are often the first-line treatment for mild to moderate pain due to their more favourable safety profile compared to opioids. Adjuvant medications, including gabapentinoids, antidepressants, and local anaesthetics, can enhance pain relief when combined with primary analgesics. These agents act on different pain pathways, reducing the need for opioids and thereby lowering associated risks [9]. Research supports that multimodal analgesia, which combines different classes of analgesics, can provide superior pain relief while minimizing side effects typically caused by higher doses of a single medication. For example, combining NSAIDs with opioids enhances overall pain control and allows for lower opioid doses, thus reducing the risk of opioid-related adverse effects [10]. Non-pharmacologic interventions also play a vital role in pain management. Physical therapy, for instance, assists in pain relief through movement and rehabilitation, particularly for chronic pain conditions [18]. CBT is another effective intervention, helping patients manage pain by altering their perceptions and responses to pain stimuli. Moreover, alternative therapies, such as acupuncture, massage, relaxation techniques, meditation, and breathing exercises, can provide significant relief and enhance the overall pain management experience. Simple methods, like heat or cold, also manage acute pain by reducing inflammation and improving blood circulation [18]. Multimodal pain management emphasizes the simultaneous use of multiple analgesics that target different pain pathways. This approach aims to optimize pain relief while reducing opioid dependency. By integrating pharmacologic and non-pharmacologic strategies, healthcare providers can tailor pain management to each patient's individual needs, enhancing both efficacy and safety [10]. Evidence suggests that multimodal approaches result in better pain control, lower opioid consumption, and fewer side effects. Combining regional anaesthesia with systemic analgesics has been shown to reduce postoperative pain and opioid use in surgical patients. This approach also allows personalized treatment based on the patient's pain profile, preferences, and clinical condition, improving overall patient satisfaction and outcomes [10].

Challenges and barriers

Optimizing pain management in EDs presents several challenges and barriers that must be addressed to ensure effective and safe patient care. These challenges can be classified into operational, clinical, and patient-related factors [19]. Operational challenges are a major concern in EDs. Time constraints are paramount; the fast-paced environment often limits thorough pain assessment and management time. Clinicians must make quick decisions while balancing the need for timely pain relief with the risks associated with certain analgesics [20]. This urgency can result in rushed assessments and potentially inadequate pain management. Additionally, staffing and resource limitations further complicate the situation. Many EDs operate with limited personnel and may lack the resources to implement comprehensive pain management strategies. This can affect the availability of non-pharmacological interventions and hinder the ability to provide individualized care tailored to each patient’s needs [20]. Clinical challenges also play a significant role. Variability in pain assessment and management among clinicians and different EDs can lead to inconsistent patient care. This inconsistency can result in some patients receiving inadequate treatment, while others may be overmedicated. Standardizing pain assessment and management protocols could help address this issue [21]. Furthermore, the risk of opioid misuse and addiction remains a critical concern. The opioid crisis has heightened awareness of the dangers associated with opioid prescriptions. Clinicians must carefully balance the need for effective pain management with the risks of opioid use, which can lead to addiction and other adverse outcomes. This balance adds another layer of complexity to pain management in the ED [21]. Patient-related challenges also significantly impact pain management. Many patients enter the ED with expectations of rapid pain relief, creating pressure on clinicians to prescribe opioids. However, effective communication about the risks and benefits of different analgesics is essential but can be challenging in the fast-paced ED setting [22]. Clinicians must navigate these expectations while providing appropriate and safe pain management options. Cultural and psychological factors further complicate the situation. Patients’ beliefs, past experiences with pain, and psychological conditions can influence their perception of pain and response to treatment. Addressing these factors requires a patient-centred approach that considers the individual’s unique circumstances, including their cultural background and psychological state [22]. Challenges and barriers to effective pain management are detailed in Table 2.

**Table 2:** Challenges and barriers to effective pain management

Challenge/barrier	Description	Impact on pain management	Potential solutions
Opioid overuse and dependence [23]	High risk of addiction and misuse associated with opioid prescriptions, leading to increased regulatory scrutiny and hesitation in prescribing	Limits the use of effective pain relief options, especially for severe pain	Implementing opioid stewardship programs, exploring non-opioid alternatives, and educating patients on safe use
Lack of standardized protocols [24]	The absence of uniform pain management protocols across different emergency departments leads to inconsistent care	Inconsistent pain assessment and treatment across providers, resulting in variable patient outcomes	Developing and implementing standardized pain management guidelines for emergency care settings
Underreporting of pain [25]	Patients, particularly vulnerable populations (e.g., elderly, paediatric, non-native speakers), may underreport their pain due to communication barriers or fear	Inadequate pain relief and delayed diagnosis of underlying conditions	Improved communication tools, regular pain assessments, and training for healthcare providers in pain recognition
Time constraints in emergency departments (EDs) [26]	Emergency department (ED) personnel often face time pressure, reducing the ability to provide thorough pain assessments and individualized care	Leads to rushed assessments, insufficient pain relief, or over-reliance on quick fixes like opioids	Streamlining assessment tools, integrating rapid pain management protocols, and triaging based on pain severity
Fear of respiratory depression [27]	Concerns about respiratory depression limit the aggressive management of pain, especially with opioids, in acute settings	Suboptimal pain control in patients at risk for complications	Training in risk management, close monitoring of high-risk patients, and using multimodal analgesia to reduce opioid reliance
Cultural and socioeconomic barriers [28]	Cultural beliefs, socioeconomic status, and access to healthcare can affect how pain is perceived, reported, and treated	Miscommunication or misunderstanding of pain severity, limited access to adequate pain care for underprivileged populations	Cultural competency training, equitable access to care, and considering social determinants in pain management plans
Overcrowding in EDs [29]	Overcrowding in emergency departments can delay timely pain management interventions	Delayed treatment, patient dissatisfaction, and prolonged pain in acute scenarios	Improving ED workflows, prioritizing pain relief in triage systems, and incorporating fast-acting interventions
Limited use of non-pharmacological methods [30]	Lack of awareness or training in non-pharmacological approaches such as physical therapy, acupuncture, or psychological support for pain management	Over-reliance on medications, missing out on effective adjunct therapies that could improve patient outcomes	Increasing training and awareness, integrating multidisciplinary approaches to pain management in the ED
Patient expectations [31]	Patients may expect immediate pain relief, often through medications, putting pressure on providers to prescribe potent analgesics like opioids	This may lead to over-prescription of opioids or dissatisfaction with slower-acting alternatives	Setting realistic expectations, educating patients on pain relief timelines, and promoting multimodal pain management
Inadequate pain assessment tools [32]	Existing pain assessment tools may not fully capture the patient’s pain experience, especially in cases of chronic or complex pain syndromes	Misunderstanding of pain severity, leading to under-treatment or overtreatment	Developing more nuanced pain assessment tools, particularly for non-verbal patients and those with complex conditions

Innovations and emerging practices

Innovations and emerging practices in pain management, especially within EDs, are significantly driven by technological advancements, new medications and techniques, and ongoing research. These developments aim to improve patient outcomes and address the complexities of managing acute pain more effectively [33]. Technological advancements have dramatically transformed pain assessment and management in EDs. Electronic health records (EHRs) are crucial in this evolution, offering comprehensive patient data that informs clinical decisions. Integrated decision support systems within EHRs help healthcare providers choose appropriate analgesics based on patient history, current medications, and pain severity [34]. These systems also enable timely pain reassessment, allowing for adjustments to treatment plans as the patient’s needs change. Additionally, recent innovations in pain assessment tools, such as artificial intelligence (AI), are enhancing the objectivity of pain measures. AI-driven tools can analyse physiological data and behavioural indicators, improving the accuracy of pain detection, particularly in patients who cannot self-report effectively. Automated pain tracker devices are also being piloted in EDs, allowing patients to report pain levels in real time, which enhances documentation and management [35]. Developing new medications and techniques is another key area of innovation in pain management. The pharmaceutical field is seeing the emergence of new analgesics that provide effective pain relief while minimizing the risks associated with traditional opioids [36]. These new medications target specific pain pathways, offering safer alternatives for managing acute pain in ED settings. Non-pharmacologic techniques are also gaining attention. Innovations such as movement-based pain assessments and CBT are being explored to complement traditional pain management strategies. These approaches empower patients to manage pain through physical activity and psychological support, ultimately reducing reliance on medications [36]. Ongoing research and evidence-based practices are critical in shaping pain management strategies in EDs. Recent studies highlight the effectiveness of multimodal pain management approaches that combine pharmacologic and non-pharmacologic interventions. Research supports using non-opioid analgesics as first-line treatments for many acute pain conditions, emphasizing the importance of comprehensive pain assessments to guide treatment decisions [37]. Furthermore, implementing best practices is essential for optimizing patient outcomes. Guidelines recommend regular pain assessments, ideally every 15 minutes for patients in severe pain, and the use of validated pain scales to gauge pain intensity accurately. Training for ED staff on the latest pain assessment tools and management strategies is crucial to ensure adherence to these best practices [37]. Innovations and emerging practices in pain management are detailed in Table 3.

**Table 3:** Innovations and emerging practices in pain management

Innovation/emerging practice	Description	Applications in pain management	Advantages	Challenges/considerations
Personalized pain management [38]	Tailoring pain management strategies to individual patient profiles, including genetics, pain tolerance, and medication response	Chronic pain conditions, post-operative care, opioid management	Higher efficacy, reduced side effects, improved patient outcomes	Requires advanced diagnostic tools, high cost, and complex implementation
Telemedicine for pain management [39]	Remote monitoring and consultation for pain management, allowing access to care in rural or underserved areas	Chronic pain management, follow-up care after surgeries	Increased access to specialists, convenience for patients, lower cost	Limited by internet access and technology literacy, regulatory and reimbursement issues
Artificial intelligence (AI) and machine learning in pain prediction [40]	Using AI algorithms to predict pain levels based on vital signs, patient history, and facial expressions	Real-time pain assessment, predicting post-surgical pain severity, personalizing opioid doses	Enhanced pain prediction accuracy, earlier interventions, reduced subjectivity in pain assessments	Requires large data sets, privacy concerns, and potential biases in AI algorithms
Multimodal analgesia optimization [41]	Further development of multimodal approaches that combine various pain relief techniques (e.g., pharmacological, physical therapy, psychological support)	Post-surgical pain, trauma, chronic pain	Reduces opioid use, more comprehensive pain relief, minimizes side effects	Requires collaboration between specialities, complex dosing regimens
Non-opioid analgesic alternatives [42]	Development of new non-opioid pain medications such as non-opioid pain (NOP) receptor agonists, transient receptor potential vanilloid 1 (TRPV1) antagonists, and sodium channel blockers	Post-surgical pain, acute pain, chronic pain	Reduced risk of addiction, lower chance of respiratory depression	Some alternatives are still in the early stages of clinical trials, with unknown long-term effects
Virtual reality (VR) for pain distraction [43]	The use of immersive VR environments to divert attention away from pain has proven effective in acute settings like burn treatments and physical therapy for chronic pain	Acute pain, procedural pain (e.g., wound care), physical therapy for chronic pain	Non-invasive, can be tailored to patient preferences, highly effective for reducing perception of pain	High cost of VR systems, not universally applicable for all patients, potential motion sickness
Genetic testing for pain sensitivity [44]	Using genetic testing to identify variations in pain perception and opioid metabolism, allows for personalized pain management strategies	Chronic pain, surgical pain, opioid prescription	Personalized pain relief, reduced risk of opioid overuse or underuse	Expensive, limited availability, ethical concerns around genetic information
Targeted drug delivery systems [45]	Innovations in drug delivery systems include transdermal patches, nanoparticle carriers, or extended-release formulations for localized and sustained pain relief	Chronic pain, cancer pain, post-surgical pain	Prolonged pain relief, reduced systemic side effects, more targeted pain control	Development and manufacturing costs, complex regulatory approvals
Neuromodulation techniques [46]	Advanced techniques such as spinal cord stimulation, deep brain stimulation, and peripheral nerve stimulation modulate pain signals directly in the nervous system	Neuropathic pain, chronic pain, cancer pain	Effective for severe, intractable pain, non-pharmacological	High cost, requires specialized training and equipment, invasive procedures
Stem cell therapy for pain relief [47]	Experimental use of stem cells to regenerate damaged tissue or modulate the inflammatory response, potentially reducing chronic pain	Chronic joint pain, back pain, arthritis	Potential for long-term relief, reduced reliance on medications	Still in the experimental phases, ethical concerns, and high cost, require further research

Recommendations for optimization

Optimizing pain management in EDs is critical for improving patient outcomes and satisfaction. The following recommendations address key areas for enhancement, including pain assessment, medication practices, multimodal approaches, and systemic issues [48]. Effective pain management begins with improved pain assessment. ED staff should receive enhanced training to develop the skills and knowledge necessary for accurate pain evaluation and management [49]. This training can include regular sessions on pain assessment techniques and tools, utilizing case studies and role-playing to improve understanding and application of pain management strategies. Standardized pain assessment tools, such as the Numerical Rating Scale (NRS) or the Wong-Baker FACES Pain Rating Scale, should be adopted to ensure consistency and accuracy. These tools should be integrated into EHR systems to facilitate streamlined documentation and tracking [49]. Optimizing medication practices is another crucial area. Developing and adhering to standardized protocols can enhance medication practices and improve pain management outcomes [50]. Evidence-based protocols should outline the use of non-opioid and opioid analgesics according to pain severity, with regular updates to reflect the latest research and guidelines. Educating ED staff on safe opioid use and alternative options is essential to mitigate the risk of opioid misuse while ensuring effective pain relief. Educational sessions should emphasize the risks associated with opioids, the importance of prescribing the lowest effective dose for the shortest duration, and the appropriate use of non-opioid alternatives [50]. Integrating multimodal approaches can improve pain relief and reduce opioid dependence. Utilizing a combination of analgesics, such as NSAIDs and acetaminophen, alongside adjunct therapies like physical therapy, acupuncture, or CBT, can offer a more comprehensive pain management strategy. Developing care pathways that incorporate pharmacologic and non-pharmacologic modalities for specific conditions ensures a holistic approach to pain management [11]. Addressing systemic issues is also vital for supporting effective pain management practices. Enhancing staffing and resource allocation can ensure sufficient personnel and resources to support pain management initiatives [51]. This may involve evaluating current staffing levels and patient volumes to determine the needs for additional support and advocating for increased funding or resources for pain management. Improving patient communication and education can also empower patients to be more involved in pain management. Developing educational materials that explain pain management options, potential medication side effects, and the importance of reporting pain levels, as well as fostering open communication between patients and healthcare providers, can contribute to a safer and more effective ED experience [51]. EDs can significantly improve their pain management practices by implementing these recommendations. Enhanced pain assessment, standardized medication protocols, multimodal approaches, and systemic support will improve patient outcomes and create a safer, more effective overall ED experience [52]. Recommendations for optimizing pain management practices are detailed in Table 4.

**Table 4:** Recommendations for optimization of pain management practices

Recommendation	Description	Implementation strategies	Expected outcomes	Challenges
Standardization of pain protocols [53]	Developing and implementing standardized pain assessment and management protocols across all emergency departments	Training healthcare providers on standardized guidelines, regular audits, and updates based on emerging evidence	Consistent pain relief, reduced variability in care, improved patient outcomes	Resistance to change, varied resources across healthcare facilities
Enhanced education and training [54]	Providing ongoing education to healthcare providers on pain management techniques, including non-pharmacological approaches	Incorporating pain management modules, regular workshops, and simulations in medical and nursing education	Better pain assessment skills, more effective use of multimodal analgesia, reduced opioid reliance	Time and resource investment, ensuring all staff participate
Promotion of multimodal analgesia [55]	Encouraging multimodal approaches combining pharmacological and non-pharmacological methods for comprehensive pain relief	Protocols promote combining analgesics with different mechanisms, integrating physical therapy, and counselling	Reduced opioid use, improved patient satisfaction, more effective pain control	Coordination between specialities, potential increased cost in the initial implementation
Patient-centred pain management [56]	Focusing on individualized pain management strategies tailored to each patient’s needs and preferences, including cultural considerations	Involving patients in decision-making, using comprehensive pain assessments, and considering psychosocial factors	Improved patient satisfaction, better adherence to pain management plans, reduced underreporting of pain	Time-consuming, requires thorough assessments, potentially difficult to implement with high patient volumes
Improved use of non-pharmacological methods [57]	Increasing the adoption of non-pharmacological approaches like physical therapy, cognitive-behavioural therapy, and acupuncture	Integrating multidisciplinary pain teams, improving access to alternative therapies, and educating patients on their benefits	Holistic pain management, reduced medication side effects, long-term improvement in pain control	Lack of availability, higher upfront costs, patient reluctance to adopt non-traditional methods
Optimizing opioid prescribing practices [36]	Reducing opioid misuse by implementing opioid stewardship programs and promoting non-opioid alternatives for pain relief	Regular monitoring of opioid prescriptions, education on safe prescribing, and setting clear guidelines on opioid use	Lower rates of opioid dependency and misuse, better patient outcomes, safer prescribing practices	Resistance from providers accustomed to opioid prescribing, patient expectation of immediate pain relief
Telemedicine for pain management [58]	Expanding telemedicine to manage chronic pain patients and provide timely follow-ups	Developing telemedicine infrastructure, training providers on virtual consultations, and ensuring accessible technology	Increased access to care, reduced patient travel burden, ongoing pain monitoring, and management in underserved areas	Technical challenges, privacy concerns, insurance and reimbursement limitations
Improved pain assessment tools [35]	Developing more nuanced and comprehensive pain assessment tools to better capture the complexity of pain experiences	Incorporating advanced pain scales, using artificial intelligence (AI)-driven pain detection tools, and ensuring frequent reassessment	Better understanding of pain severity, improved treatment planning, more tailored pain management strategies	Development and implementation costs, ensuring healthcare provider adoption of new tools
Addressing cultural and socioeconomic barriers [59]	Developing culturally sensitive pain management strategies and addressing socioeconomic factors that limit access to care	Training in cultural competence, ensuring equitable access to pain management resources, and addressing social determinants	Reduced health disparities, improved pain management outcomes for diverse populations	Resource-intensive requires systemic changes to address social and economic barriers
Patient and caregiver education [60]	Providing thorough education to patients and caregivers on pain management options, safe medication use, and the role of non-drug interventions	Creating patient-friendly educational materials, incorporating pain education into discharge planning, and caregiver support	Better pain control, higher patient satisfaction, reduced risk of medication misuse	Ensuring that educational materials are accessible and understandable to all patients, requiring time for proper education

## Conclusions

In conclusion, effective pain management in EDs is crucial for alleviating patient suffering and improving clinical outcomes in high-pressure settings. This review has highlighted the critical role of pharmacologic and non-pharmacologic strategies in managing acute pain while addressing the unique challenges faced in the emergency care environment. By assessing current practices, identifying areas for improvement, and offering evidence-based recommendations, this review underscores the need for ongoing evaluation and optimization of pain management protocols. Implementing standardized, evidence-based practices and addressing operational and clinical challenges can significantly enhance the quality of care provided in EDs. Ultimately, these improvements ensure that all patients receive timely and effective pain relief, fostering better patient experiences and more favourable health outcomes. As emergency care continues to evolve, ongoing research and innovation will be essential in refining pain management strategies and addressing the dynamic needs of patients in these critical settings.
